# Lessons Learned from Community Contact Tracing of Women Disengaged from an Urban Prevention of Mother-to-Child Transmission Program in Uganda

**DOI:** 10.1007/s10995-026-04229-4

**Published:** 2026-02-18

**Authors:** Ellon Twinomuhwezi, Agnes N. Kiragga, Grace Banturaki, Marion Achieng, Juliet Nampala, Irene Bagaya, Joanita Kigozi, Barbara Castelnuovo, Rohan Hazra, Constantin T. Yiannoutsos, Kara K. Wools-Kaloustian

**Affiliations:** 1 Infectious Diseases Institute, College of Health Sciences, Makerere University, P.O. Box 22418, Kampala, Uganda; 2 National Institute of Child Health and Human Development (NICHD), National Institutes for Health, Bethesda, MD USA; 3 IRCCS Neuromed, Doctors with Africa CUAMM, Bari, Italy; 4 Fairbanks School of Public Health, Indiana University, Indianapolis, IN USA; 5 School of Medicine, Indiana University, Indianapolis, IN USA

**Keywords:** Prevention of mother-to-child transmission, Disengagement, Tracing, Outreach

## Abstract

**Purpose:**

Community tracing for disengaged pregnant and post-partum women with HIV(PPWH) shows promise, yet few studies detail the practical challenges of tracing, particularly in the Prevention of Mother-to-Child Transmission programs (PMTCT). Standard guidelines and evidence-based approaches remain lacking. We share lessons from our experiences in tracing disengaged PPWH in an urban PMTCT program in Uganda, where nurse counselors joined outreach teams to trace lost PPWH women via calls and home visits.

**Description:**

The parent study was a prospective case–control study conducted in Uganda at six urban clinics. We integrated study nurse counsellors into the program’s outreach team to support the participant tracing activities. A team of outreach workers, including study nurse counsellors, traced the disengaged women through telephone calls and home visits.

**Assessment:**

Experiences encountered and lessons learned by the nurse-counselors encompassed the following domains: non-disclosure, stigma, and cultural practices; sex of the tracer (outreach worker); client mobility; incorrect locator information; community-specific names; deregistration of phone lines; and non-disclosure of exposure status in infants whose mother had died.

**Conclusion:**

Experienced outreach workers should complete locator forms, multiple phone number should be collected and those of trusted confidents should be identified, psychosocial issues should be addressed early, outreach team members should come from the local community, locator information should be kept up to date, an electronic system with the capacity to store GPS coordinates should be considered, gender match tracers to the client, local protocols should be developed for the disclosure of exposure status for infants whose mothers have died.

## Significance

Attrition from PMTCT programmes remains a major barrier to eliminating paediatric HIV, and there's alack of standardised, evidence-based guidelines for tracing women who disengage. This paper off erspractical insights from community-based contact tracing of disengaged pregnant and postpartumwomen with HIV in urban Uganda, highlighting challenges such as stigma, gender dynamics, clientmobility, and data gaps. It also shares adaptive strategies used by nurse counsellors and outreachworkers, providing a framework for eff ective tracing protocols. These lessons are fundamental tostrengthening PMTCT, improving outcomes for women and infants, and supporting global eff ortstowards an AIDS-free generation.

## Introduction

Without antiretroviral treatment (ART), pregnant women living with Human Immunodeficiency Virus (HIV) have a 15–45% risk of transmitting the virus to their unborn children during pregnancy, at delivery, or while breastfeeding.(Joint United Nations Programme on HIV/AIDS (UNAIDS), 2016; The World Health Organization (WHO), 2019; WHO, 2021) Global efforts to expand Prevention of Mother-to-Child Transmission (PMTCT) programs have resulted in notable progress, with 85% of pregnant women living with HIV receiving ART as of 2021.(The Joint United Nations Programme on HIV/AIDS (UNAIDS),

2018) However, despite the successful implementation of PMTCT programs to prevent new HIV infections in children, the PMTCT cascade still faces high attrition rates among pregnant and post-partum women. The PMTCT cascade, also known as the prevention of mother-to-child transmission continuum of care, is the sequential series of steps that a pregnant woman living with HIV and her infant must complete to effectively prevent vertical transmission, from initial HIV testing and antiretroviral therapy initiation to postpartum follow-up and final infant diagnosis. (Castelnuovo et al., 2017; Hamilton et al., 2017; Myer & Phillips, 2017) This poses a serious risk, as infants born to mothers who disengage from care are more likely to acquire HIV. (Estill et al., 2018; HIVinfo@NIH.gov, 2024; Izudi et al., 2018; Kiragga et al., 2021) Furthermore, only half of HIV-exposed children receive early diagnostic testing via Deoxyribonucleic acid Polymerase chain reaction (DNA PCR) within the first three months of life, as recommended by the Joint United Nations Programme on HIV/AIDS (UNAIDS) and the World Health Organization (WHO).(Joint United Nations Programme on HIV/AIDS (UNAIDS), 2016, 2017; World Health Organization, 2018).

Against this background, PMTCT programs are exploring options such as physically tracing pregnant and post-partum women with HIV (PPWH) with outreach teams to close gaps and decrease the risks of loss to follow-up, while maintaining confidentiality and enhancing re-engagement rates.(Vogt et al., 2015) Tracing disengaged women in PMTCT programs is essential for many reasons. Disengagement often results in untreated maternal HIV progression and poorer health outcomes for HIV-exposed infants.(Bailey et al., 2013; HIVinfo@NIH.gov, 2024; Izudi et al., 2018; WHO, 2021) (Larsson et al., 2015). Tracing supports reintegration of women into care, ensuring access to ART, infant testing (such as early DNA PCR), and infant immunizations.(HIVinfo@NIH.gov, 2024; WHO, 2021). Keeping women engaged in PMTCT programs advances global efforts to eliminate pediatric HIV and fosters sustainable health systems by reducing long-term treatment costs and new infections.(HIVinfo@NIH.gov, 2024; Larsson et al., 2015; Sawesi et al., 2016; Vogt et al., 2015).

Barriers to PMTCT retention are well-documented, including transportation issues, stigma, disclosure concerns, poverty, and strained relationships between patients and healthcare providers. (Darak et al., 2012; Gourlay et al., 2013; Kiragga et al., 2021; Mutabazi et al., 2017; Nalubega et al., 2021) Women face unique challenges, such as gender- based violence and fears of non-disclosure. While much research has explored why women disengage from PMTCT, less attention has been given to how to find and re-engage them. Studies from Malawi, Zimbabwe, Kenya, and Côte d'Ivoire have demonstrated the promise of tracing in improving retention.(McMahon et al., 2017; Rawizza et al., 2015; Teasdale et al., 2022) A study in a Ugandan fishing community found higher retention rates with physical contact compared to mobile reminders. At 12 months, retention rates were 91.5% in the physical tracing group and 82.1% in the mobile reminder group.(MoH, 2009) Similarly, a Zimbabwean study, across more than ten public health institutions, found that tracing was vital in maintaining patient retention. The results showed that overall retention rates at 6, 12, 24, and 36 months were higher in the 2012–2015 cohorts compared to the 2007–2010 groups, which did not include tracing exercises. The study emphasized the need to strengthen active patient tracing mechanisms to improve retention, especially for those lost to follow-up.(MoH, 2011).

One retrospective study in southwestern Uganda found that recording a phone number in a patient's medical file was a key factor for successful tracing, with a 10% higher tracing success rate for those with recorded phone numbers than those without. (National AIDs and STI Control Program (NASCOP), 2018) However, few other studies examine tracing processes and practical challenges, especially for women disengaged from PMTCT programs. No standardized, evidence-based guidelines exist internationally for re-engaging this group. (Gourlay et al., 2013; Larsson et al., 2015; Mutabazi et al., 2017; Sawesi et al., 2016) Active community tracing must be sensitive to local norms, socioeconomic conditions, cultural practices, and the surrounding risks of gender-based violence. Yet, shared strategies and lessons learned across programs could enhance outreach effectiveness in the postpartum period.(Darak et al., 2012; McMahon et al., 2017; Mutabazi et al., 2017; Nalubega et al., 2021) This paper examines the processes, experiences, and challenges encountered during contact tracing for disengaged PPWH. The tracing activities described in this paper were part of a prospective study, “Tracing Non-Retained HIV Positive Pregnant Women enrolled in Option B + and ascertaining their babies’ outcomes” (STEPIWSE), which aimed to assess the outcomes among women who initiated ART for PMTCT, including those lost to follow-up.(Kiragga et al., 2021; Rawizza et al., 2015) Due to the scarcity of data on PMTCT-specific tracing, this paper focuses on the operational insights and lessons learned from re-engagement efforts in this critical population.

## Methods

### Ethical Considerations

The study received approval from the Joint Clinical Research Center Institutional Review and Ethics Committee (JCRC-REC), the Uganda National Council of Science and Technology (UNCST) (Ref: HS35ES), and the Indiana University Institutional Review Board. All enrolled women provided written informed consent for study participation for themselves and their infants.

### Study Design and Study Site

The parent study was a prospective case–control study conducted in Uganda at six Kampala Capital City Authority (KCCA) clinics supported by the Infectious Diseases Institute (IDI), Makerere University, and the Uganda Centers for Disease Control and Prevention's President’s Emergency Plan for AIDS Relief (CDC-PEPFAR), the HIV care and treatment partner. These sites were chosen because they offer comprehensive PMTCT services, including two key interventions for newly diagnosed pregnant women with HIV: same-day ART initiation and pre-ART adherence counseling. (Humphrey et al., 2024; Teasdale et al., 2022) During the study period, the first-line regimen recommended by the Ugandan National Treatment Guidelines was a three-drug regimen of tenofovir, lamivudine, and efavirenz (TDF, 3TC, EFV), along with cotrimoxazole prophylaxis. (MoH, 2009, 2011 National AIDs and STI Control Program (NASCOP), 2018) Furthermore, there was little variation in how care was provided across the clinics, as they all followed these guidelines, including the protocol for HIV viral load testing, which recommended monitoring at six months and annually afterward.(National AIDs and STI Control Program (NASCOP), 2018).

### Study Population

Women living with HIV (WLHIV) who were 18 years or older, initiated ART during pregnancy (confirmed by a positive pregnancy test or physical exam evidence of pregnancy), and were 6–12 weeks post-partum were eligible for the study. Women known to have died or been transferred to other HIV care facilities were excluded. Index cases were women who had disengaged from care. A woman was considered disengaged if she had not returned to the health care facility for her clinic appointment. Within the program's standard operating procedures, clients were typically classified as lost to follow-up (LTFU) if they had missed a scheduled appointment by 90 days. To align with this operational definition and ensure we captured a stable cohort for tracing, we defined a woman as disengaged if she had not returned to the healthcare facility for a clinic appointment. Women whose delivery occurred before they disengaged were considered to have delivered in care, while those whose infants were born after they disengaged were classified as delivered out of care. The controls were women who had been retained in care. A woman was defined as retained if she was actively attending the health care facility with at least one clinic encounter within 90 days of data extraction, and she had also attended her 6-week follow-up visit within 10 weeks of the estimated delivery date. Infants born to disengaged women were referred to as disengaged babies, whereas infants born to women who remained in care were called retained babies.

### Standard of Care

During enrollment in the antenatal care unit (ANC), all pregnant women are tested for various sexually transmitted infections, including HIV.(WHO, 2021; World Health Organization, 2021) Women found to have HIV are transferred with all of their documentation from the general ANC unit to the PMTCT clinic. On the day of diagnosis, women are counseled, the results are discussed, and they are started on ART immediately. After the ART initiation visit, women are considered fully enrolled in the PMTCT program. (Flax et al., 2017; Joint United Nations Programme on HIV/AIDS (UNAIDS), 2017; World Health Organization, 2021) Throughout the process, women who wish to transfer their care to another center due to convenience or relocation are encouraged to request a formal transfer from their enrollment site, which is documented in the women’s records.

During the ART-initiation visit, counselors or mother mentors complete locator forms that include directions to the woman’s home, a sketched map, names commonly used by the woman in the community or her workplace, occupation, next of kin, all usable phone contacts, community leader contacts, ART clinic numbers, and treatment support contacts. Mother mentors are long-standing clients living with HIV who are familiar with the system and considered by the clinic staff to have positively adapted to their status. Mentors serve as lay counselors and provide support through facility non-clinical services such as patient triage, counseling, cotrimoxazole pill counts, and recording patient locator information. (DiCarlo et al., 2018; Igumbor et al., 2019; Schmitz et al., 2019) The clients are also registered in the general ART clinic registry book, a real-time record of all newly diagnosed clients who have started ART as part of the test and treat programme. (Hamilton et al., 2017; MoH, 2009, 2011). This information is entered into the Medical Record System (MRS).

After the initial visit, women are scheduled for a two-week follow-up appointment. They continue to be seen every 4 weeks throughout pregnancy. The first post-partum visit occurs at six weeks after delivery, when the infant has blood taken for an HIV DNA PCR test and undergoes routine immunization and growth monitoring. (HIVinfo@NIH.gov, 2024; Izudi et al., 2018; Larsson et al., 2015) As part of routine care, if a woman does not return for her scheduled appointment, the outreach team’s counsellor assigned to the Early Infant Diagnosis (EID) program is responsible for trying to contact her by phone the day after the missed visit.(Castelnuovo et al., 2017; Izudi et al., 2018; Kiragga et al., 2021; Myer & Phillips, 2017)] If the phone contact is successful, the counsellor discusses the reasons for the missed appointment and offers to reschedule. When a woman cannot be reached after several phone attempts, the outreach team begins physical tracing using the locator card information. Three months after community tracing starts, the case is recorded as LTFU if the team cannot successfully contact and re-engage the woman (Table 1).

**Table 1:** Comparison of standard of care tracing versus enhanced study tracing procedure

Feature	Standard of care clinic tracing	Study tracing procedures
Initial contact		
timing	Within 1 day of a missed scheduled appointment	Six weeks after a missed 6-week postpartum visit
Initial contact attempt	Initial phone call	Three phone attempts on different days/times
Who initiates	Call initiated by EID counsellor	Initiated by either the outreach team or the nurse counselor
Home visit		
Team composition	Clinic-based outreach team	Existing outreach team and study nurse counsellors
Visit procedures	If phone contact fails, conduct it using the locator form information	Conduct after failed phone attempts; structured approaches for different situations (woman alone, not alone, or not found)
Community entry	Relies on existing knowledge of the outreach team	Guided by study tracing SOPs
Number of attempts	Traced once	Three tracing attempts were made
Objective	To reschedule a missed appointment and ensure medication adherence	To locate, re-engage in care, and enroll the woman and her infant in the study
Terminated	After three call attempts on the same date	After three call attempts at different times on different days

Community tracing is the overall strategy that includes all efforts to find disengaged clients outside the health facility. This involves both phone calls and in-person outreach. In-person outreach specifically means outreach workers visiting a client's last known address or community to locate them. Therefore, physical tracing is the face-to-face, field-based part of the broader community tracing approach.

### Community Tracing Procedures

Most outreach team members are native to the community and have a proven track record of interacting with local organizations and leaders, including Village Health Teams (VHTs), religious leaders, chairpersons, local council representatives, and women's groups within the community.(DiCarlo et al., 2018; Fayorsey et al., 2019). The outreach team included five lay counselors and mother mentors, all with secondary education and certified training in HIV counseling, PMTCT protocols, and community engagement strategies. Their training focused on ethics, confidentiality, and handling sensitive disclosures. Before joining the outreach team, nurse counselors completed a structured orientation on the study's goals, ethical principles of community engagement (including confidentiality and informed consent), and tracing procedures. They then received practical training from the outreach team on locating women, understanding religious and cultural influences on HIV treatment and disclosure, and maintaining confidentiality during outreach. Before starting study tracing activities, nurse counselors were integrated into the outreach team. This integration was done to ensure community acceptance, adherence to the outreach standards, and to enhance the team's capacity for outreach efforts. Once the study tracing activities began, the combined team followed up with the disengaged women through phone calls and home visits to gather vital clinical information about both mother and baby. The team used standardized operating procedures (SOPs) for outreach to ensure a consistent approach to outreach. For example, three phone attempts were made before conducting home visits, and strict confidentiality was maintained to avoid stigma. Nurse counselors presented themselves neutrally as maternal/child health representatives until consent was obtained. To reduce distrust-related bias and ensure proper informed consent without coercion, the team followed the mother’s guidance on when to approach her for procedures, and the team would only release information after confirming the woman’s identity through trusted local community or religious leaders known to the outreach team and who have been helpful in other mother–child care health facility programs.

During the parent study, lists of women who had not attended their 6-week post-partum clinic visit by 12 weeks post-partum were generated from the Electronic Medical Record (EMR) at the selected health facility. The nurse counselors utilized the procedures outlined above to trace these women. While striving to maintain confidentiality, the team was sensitized to three potential scenarios during outreach: *Scenario 1* — The woman was located but was not alone. In this case, the team was directed to speak privately with the client and scheduled a follow-up meeting at a convenient time when she would be alone. *Scenario 2*—The woman could not be found using the available locator information. In this situation, the team engaged community informants, such as neighbors, treatment supporters, and relatives, to gather information about the woman's whereabouts while protecting confidentiality by identifying themselves as representatives of the maternal and child health program, conducting routine follow-up. If the woman was traced through the community contacts, her identity was confirmed before disclosure of any HIV related information. *Scenario 3*—The woman was found alone. In this case, in addition to trying to re-engage the woman in care, she was also educated about the study. For all women, once the nurse counselors were able to inform the women about the study in a confidential location, those willing to enroll completed the consent process and study procedures for the parent study. Study procedures are steps taken to enroll the participant during the physical tracing of PPHIV in the community. For this study, a tracing attempt was considered successful if the outreach team made direct physical contact with the disengaged woman. If the client met the inclusion criteria, they were consented and enrolled in the study, and study procedures were performed as part of enrolling the participant.(Kiragga et al., 2021).

## Results

### Tracing Outcomes

Between July 2017 and July 2018, a total of 748 women living with HIV started ART during pregnancy and participated in the PMTCT program at the participating Kampala Capital City Authority clinics. Of these, 358 disengaged women were eligible for the study and were subsequently traced. Out of these, 194 (54%) were found, with 30 declining to participate and four confirmed dead. Of the 164 women who could not be found, 81 (49.4%) had complete locator information but could not be reached, while 56 (34.1%) and 27 (16.5%) women had incorrect or incomplete locator data, respectively.(Kiragga et al., 2021).

### Experiences and Lessons Learned by the Nurse-Counsellors

The combined medical expertise and community engagement strategy were beneficial in data collection, as they ensured accuracy when explaining health-related scenarios to participants, reduced misunderstandings, enhanced trust in the study, increased participation rates, and improved response quality. Integrating study nurses with outreach teams helped address immediate concerns or referrals when needed. Experiences encountered and lessons learned by the nurse-counselors encompassed the following domains: non-disclosure, stigma, and cultural practices; sex of the tracer (outreach worker); client mobility; incorrect locator information; community-specific names; deregistration of phone lines; and non-disclosure of exposure status in infants whose mother had died. Experiences and lessons learned are detailed below for each category.

### Non-Disclosure, Stigma, and Cultural Practices

Some women had grave fears that their status would be revealed to their husbands, which was especially worrying for women who had experienced some level of intimate partner violence. This is shown by a woman who could not be tracked through her phone contacts or locator information. However, the team found her through community contacts from friends, relatives, and neighbors. Later, the team successfully located and visited her at home, where she expressed fears that her husband would find out her status. The team returned on another planned date set by the woman and carried out study procedures while her husband was not home. They ensured privacy was maintained throughout the visit, and all information was kept confidential between the study nurses and the participant. In some households, husbands make all healthcare decisions and do not provide money for transportation. This prevented women from seeking care if their husbands did not support HIV treatment or if they were unaware of their wives’ HIV status. In addition, some women choose not to attend clinic appointments out of fear of discrimination and shunning within their families or communities.

Cultural beliefs and practices acted as barriers to care for some women. Some women did not return to the clinic because of the 60-day traditional confinement period after childbirth. Others relied on prayer, traditional healers, or religious healing, often prioritizing these over evidence-based medicine, including ART. Additional barriers included beliefs that ART is witchcraft and negative past experiences with healthcare workers. The team addressed religious barriers by raising awareness and educating women about the benefits of re-engagement in care, handled family and disclosure issues through couples counseling, and responded to healthcare worker-related obstacles by providing feedback and community-based education to healthcare providers.

### Sex of the Tracer

Through community discussions and multiple observed scenes, the tracing team learned that male partners were suspicious of male tracers and contributed to couples’ insecurities. In one instance, a male tracer conducting physical tracing in the community was angrily confronted by a spouse, who warned that if he returned, he would face death threats. The client later told the tracer that her spouse believed there was a developing relationship between the two. To address this, the study deployed female tracers in communities where male–female interactions might be scrutinized or cause distrust. This change reduced gender-based tensions during outreach, improved acceptance and cooperation from male partners, and increased the safety of tracing efforts.

### Client Mobility

A portion of the disengaged women (28.1%) had relocated, which aligns with the high mobility rates reported in the area. The reasons for relocation included issues with local authorities, seeking work, and communal unpaid loans. The team used workplace contacts (such as employers or colleagues) for mobile clients to obtain updated home addresses while maintaining confidentiality.

### Incorrect Locator Information

A minority of disengaged women (13.6%) had incorrect locator information documented. Lost opportunities to update baseline locator information during the client’s facility visits throughout the antenatal phase and at or after delivery (postnatal care) were identified. Updates to the locator forms were not made after tracing activities were completed because the outreach team could only access the forms when a woman was deemed “lost to follow-up.” In one instance of incorrect information on the locator form, mother mentors could access the client through a friend who always accompanied the client to the clinic. However, most of the time, tracing was unsuccessful if the locator information was inadequate.

### Community-Specific Names

Tracers often discover that clients are called by different names in the community than those listed on the locator forms. In the community, the client was frequently identified by a name or identity linked to her children, such as Mama Sarah (mother of Sarah) or Nalongo (who gave birth to twins). This naming method was especially problematic for women with multiple children, resulting in clients being known by several different names within the community, which led to challenges for the tracers.

### Deregistration of Phone Lines

We found that it was common for clients to have numerous cell phone contacts in this setting because calling and texting within the same network is cheaper than cross-network charges. At the time of the study, the Ugandan Ministry of Information and Communication Technology (ICT) had disconnected all unregistered mobile lines, making many women no longer engaged in the program unreachable by phone. As a result, the team increased its efforts to physically trace women within the communities.

### Non-Disclosure of Infant Exposure Status When the Mother is Deceased

During community tracing, four women were discovered to have died, with three having left living infants in the care of a father or guardian. This created challenges related to disclosure. The Uganda HIV and AIDS Prevention and Control Act (2014) upholds confidentiality. However, it permits limited exceptions for public health purposes, which could be invoked in the case of an exposed infant whose mother has died.(The republic of Uganda, 2014) In the case of this study, however, to protect client confidentiality, the tracers did not reveal the deceased’s HIV status to the child’s caregiver. However, they strongly encouraged the caregiver to take the infant to the health facility for follow-up.

In summary, our tracing efforts revealed that the primary barriers to re-engagement were deeply rooted in sociocultural dynamics (stigma, gender power imbalances, cultural practices), logistical challenges (client mobility, unreliable communication infrastructure), and systemic gaps in data management (incomplete locator forms). Overcoming these barriers is not merely a logistical exercise but requires a nuanced, adaptable, and confidential approach tailored to each woman's context. Improving tracing protocols is therefore essential to address these multifactorial challenges and prevent the negative health outcomes associated with disengagement.

## Recommendations

Based on our experience with community tracing, many of these observations and subsequent recommendations are not new, but combined, they offer a framework for creating standardized tracing and outreach guidelines for PPWH. We suggest including the following components in future tracing guidelines:

- Involve individuals experienced with conducting field outreach should complete the locator forms, as they understand the challenges and nuances of tracing and are more likely to obtain adequate information for effective tracing.
- Ensure that the primary phone number listed on the tracing form belongs to the client or a trusted confidant who knows the client’s HIV status. The phone owner's relationship to the client must be documented for all numbers provided, along with whether the phone owner is a trusted confidant.
- When possible, obtain multiple mobile phone numbers as backup contacts, including all immediate family members, village health teams, and division chairpersons associated with the woman’s community of residence.
- A more intensive, pre-ART counseling targeted toward women newly diagnosed with HIV would allow staff to address barriers such as fear of disclosure, intimate partner violence, and internalized stigma; build trust; and help women develop coping strategies before they begin their treatment journey, thereby laying a stronger foundation for long-term adherence and retention.
- The outreach team should be a part of the community in which they are conducting tracing. This is an essential element in easing the community entry process.
- Implement a strategy where locator forms should be updated after each tracing attempt. If the tracing attempt was unsuccessful, inaccurate locator information should be removed from the woman’s card, and if successful, the locator card should be updated with the correct locator information.
- Female tracers are better received in such contexts because they mitigate the risk of sparking intimate partner suspicion or violence. Our results documented an instance where a male tracer was threatened by a spouse who perceived his visit as an inappropriate interaction with his wife. Female tracers can often build rapport more easily with female clients, discuss sensitive issues more openly, and navigate household dynamics with a lower risk of provoking hostility, thereby ensuring both tracer and client safety."
- Develop and utilize an electronic locator documentation system that can be transferred from one tracer to another should be developed to facilitate communication and improve the likelihood of locating a disengaged woman.(Sawesi et al., 2016) Such an electronic system could make editing easier and could be enhanced by adding GPS coordinates, route finders, and current navigation locators to simplify tracing. Using female tracers in communities where male‒female interactions may be scrutinized is advisable.
- Legal and ethical experts should collaborate with PMTCT specialists to create protocols for disclosing to caregivers the exposure status of infants whose mothers with HIV have died. This will ensure that protocols comply with local legal and ethical standards while focusing on the infant's health.

## Conclusion

The implementation of this study has contributed to the literature in two key ways: First, our documented experiences provide rare, practical insights into the process and operational realities of tracing disengaged pregnant and postpartum women with HIV (PPWH) in an urban African setting, an area where evidence-based guidelines are notably scarce. While many studies quantify retention issues, few detail the challenges encountered during community outreach. Second, our findings go beyond identifying barriers by offering context-specific solutions. By integrating study nurse counselors into existing outreach teams, we documented actionable lessons on managing confidentiality, stigma, gender dynamics, and systemic issues like mobile network deregistration. Using dedicated study nurses, although effective for this research, might limit replicability in resource-limited settings. Nonetheless, the key lessons and protocols—such as the need for specialized training, clear SOPs, and the strategic use of female tracers—are directly applicable to improving existing government or clinic-funded outreach teams.

This study highlights areas where tracing procedures and practices could be improved to increase the number of women found and re-engaged. Based on our findings, we recommend that PMTCT programs develop standardized protocols and training for outreach, including consistent approaches to confidentiality and education on key cultural issues that may affect outreach. To ensure thoroughness and accuracy, outreach workers should be involved in collecting locator information, and electronic locator systems that can capture GPS data should be implemented. Such innovations will likely increase the number of women disengaged from PMTCT who are located and re-engaged. In light of the advancement in digital technology in Africa, there is a need to digitalize the systems used to record and track women in HIV care programs.
